# Supplementary material for: Synergistic stabilization of a double mutant in chymotrypsin inhibitor 2 from a library screen in E. coli
Source: Commun Biol. 2021 Aug 18;4:980. doi: 10.1038/s42003-021-02490-7 (PMC8373930; doi:10.1038/s42003-021-02490-7)
Supplement: Supplementary file 2 — Supplemental Information [file 42003_2021_2490_MOESM2_ESM.pdf]

Supplementary Information

Synergistic stabilization of a double mutant in chymotrypsin inhibitor 2 from a library screen in *E. coli*

Louise Hamborg[1,2], Daniele Granata[1], Johan G. Olsen[1], Jennifer Virginia Roche[1], Lasse Ebdrup Pedersen[2], Alex Toftgaard Nielsen[2], Kresten Lindorff-Larsen[1], Kaare Teilum[1#]

[1]Structural Biology and NMR Laboratory and the Linderstrøm-Lang Centre for Protein Science, Department of Biology, University of Copenhagen, Ole Maaloes Vej 5, 2200 Copenhagen N, Denmark
[2]The Novo Nordisk Foundation Center for Biosustainability, Technical University of Denmark, Kemitorvet, 2800 Kgs. Lyngby, Denmark

[#]Corresponding author
Phone: +45 35 32 20 29
Postal Address: Ole Maaloes Vej 5, 2200 Copenhagen N, Denmark
Email: kaare.teilum@bio.ku.dk

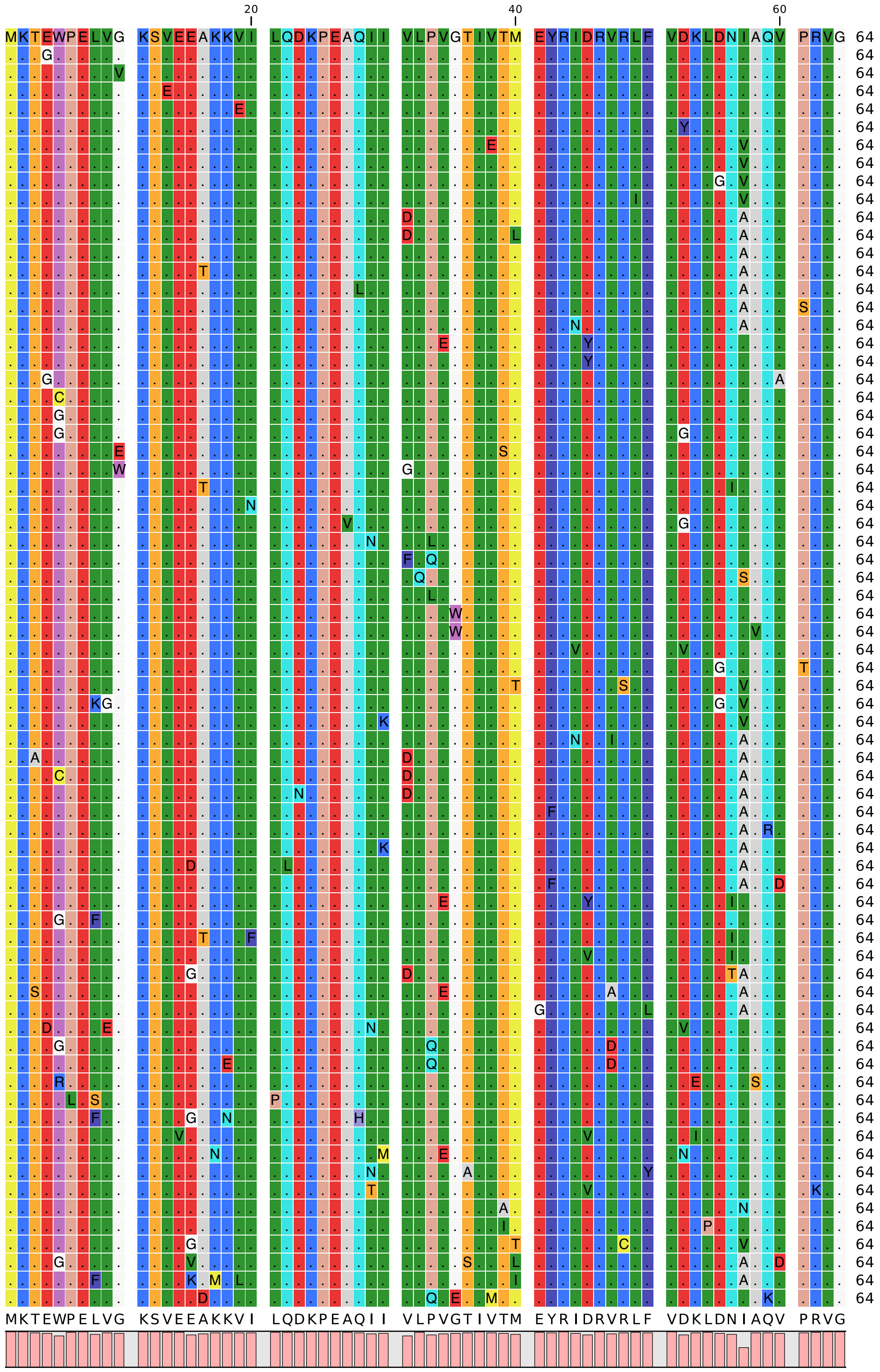

Supplementary Figure 1 Alignment of 71 CI2 sequences selected using the folding sensor. The wild-type sequence is shown in the top row. In other rows only amino acid residues that differ from the wild-type sequence are shown as letters. Below the alignment, the consensus sequence is shown on top of bars that corresponds to the level of conservation. Colours indicate amino acid types.

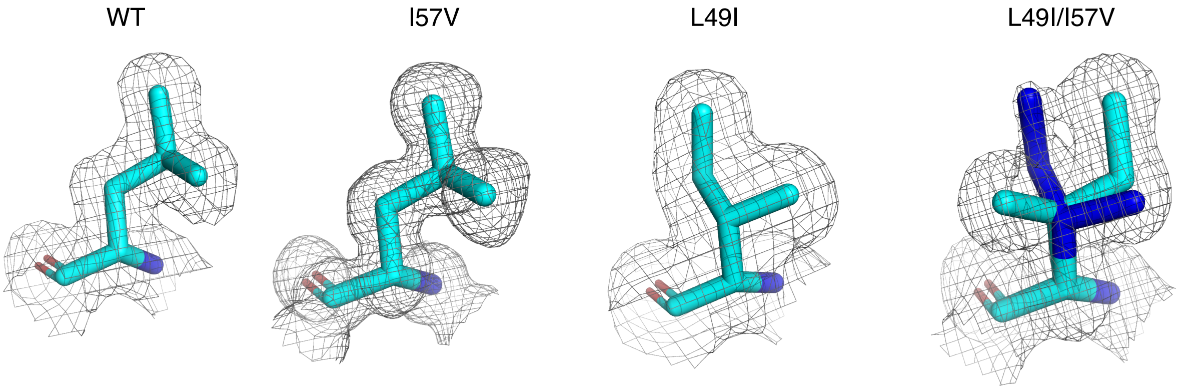

Supplementary Figure 2 Electron density around the residue at position 49 in the variant of CI2 indicated above each picture. The $2F_o$ -F_c electron density maps of the structures are plotted at 0.7σ.

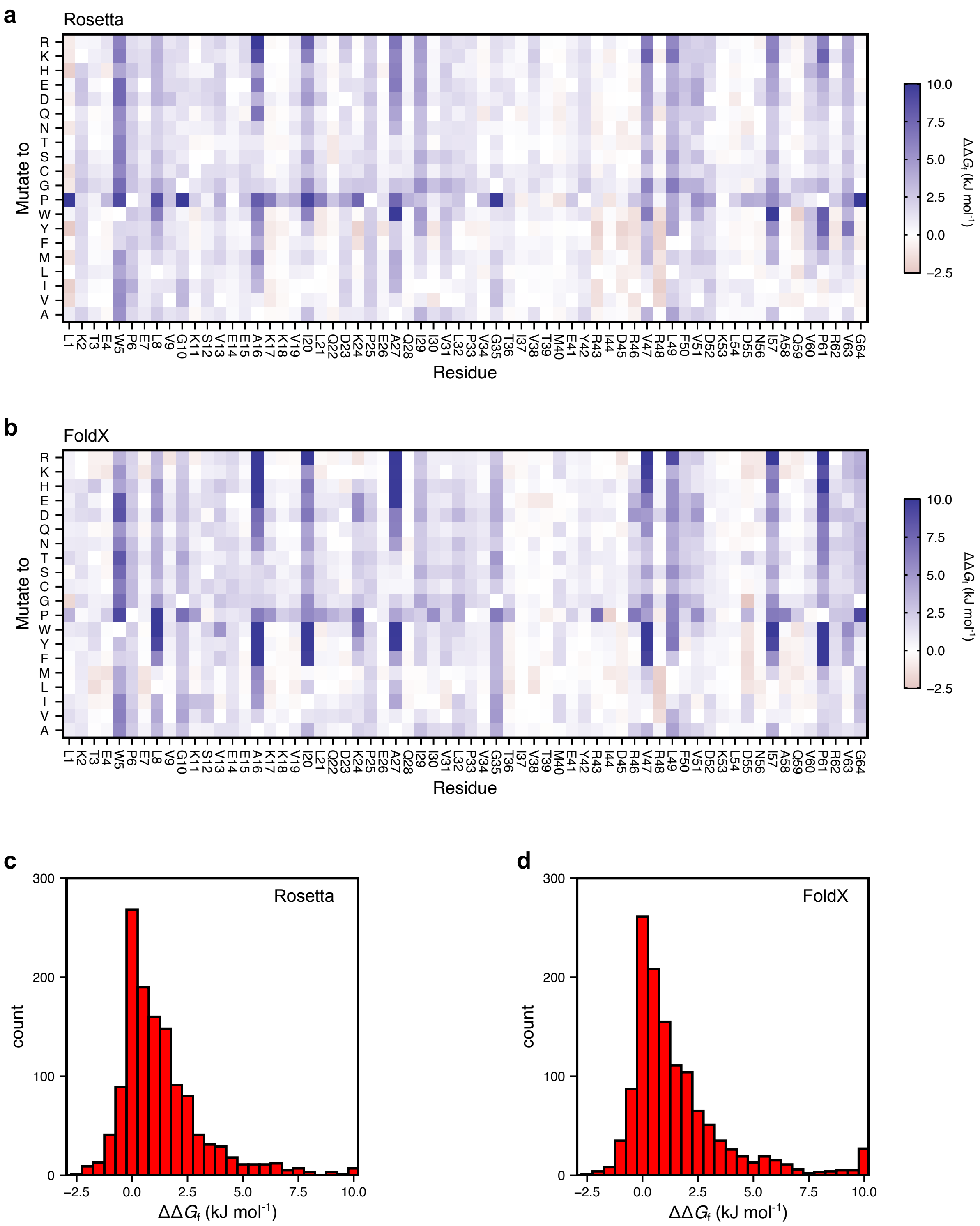

Supplementary Figure 3 Stability predictions of CI2 single point mutations. Using the original crystal structure of CI2 (PDB-code: 2CI2) we calculated the change in stability, $\Delta\Delta G_f$, for each point mutation using either (**a**) Rosetta or (**b**) FoldX. The value of the stability change is indicated with a colour scale from red (stabilizing) to blue (destabilizing) as indicated to the right of the figure. (**c-d**) The distributions of the changes in stability, $\Delta\Delta G_f$ from panel (**a**) and (**b**), respectively.

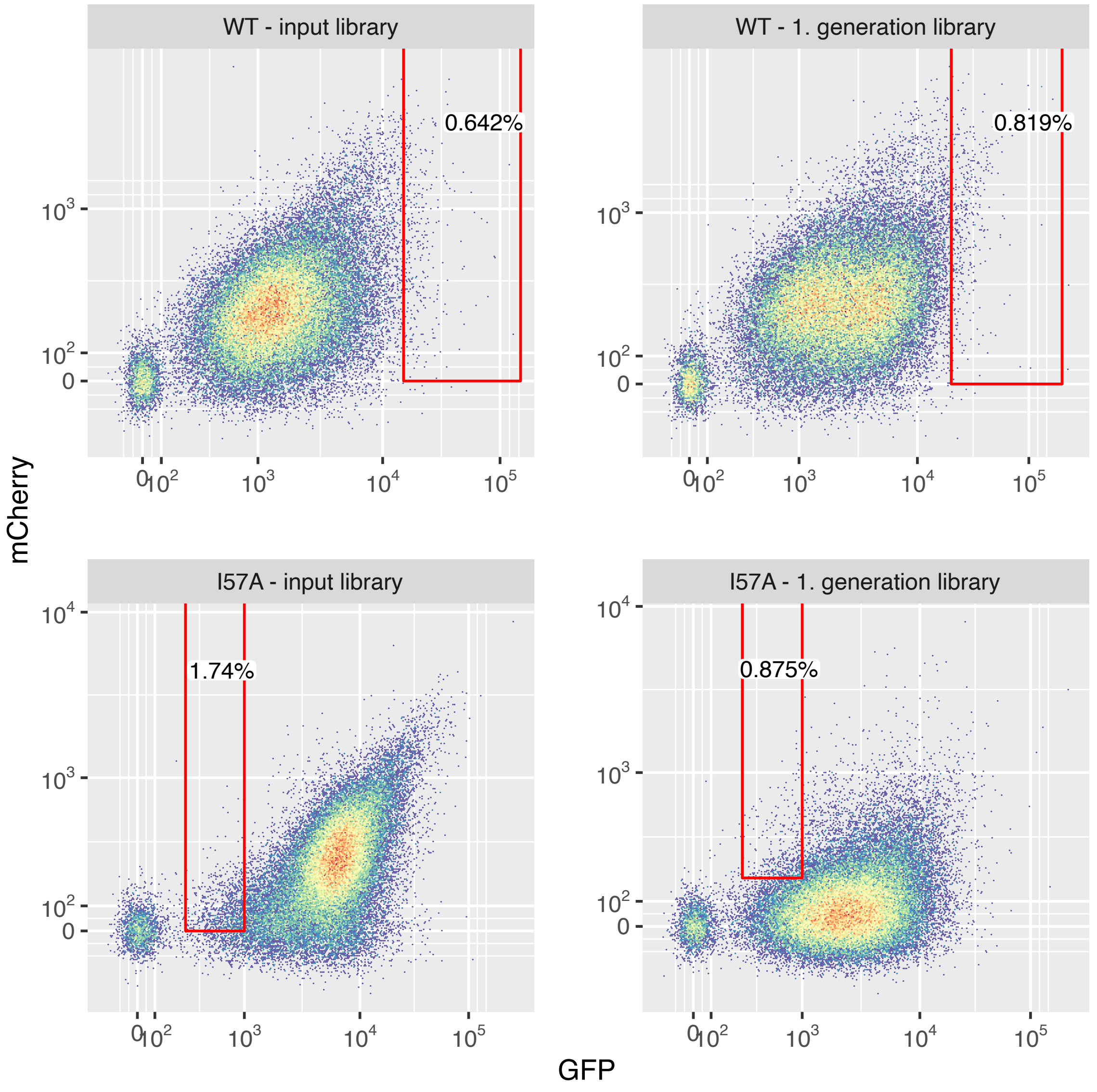

Supplementary Figure 4 Gating strategy for FACS sorting. The gates were set to collect 100.000 cells from the ~1% highest GFP fluorescence in the WT libraries or 100.000 cells from the ~1% lowest GFP fluorescence in the I57A libraries. In the second round of sorting I57A clones with mCherry signal above 300 are selected.

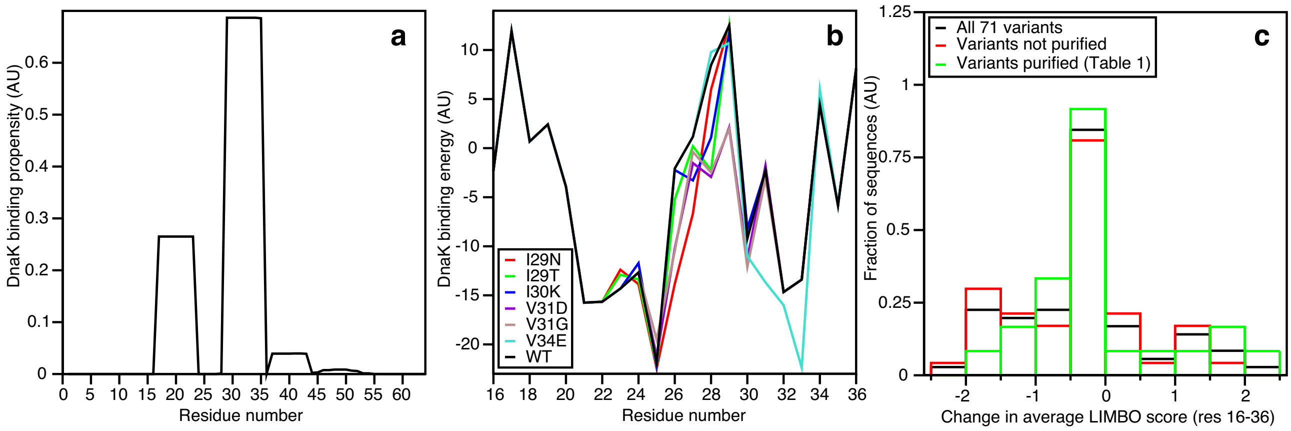

Supplementary Figure 5 Propensity for DnaK binding to CI2 as predicted by the Limbo algorithm. (**a**) Sequence dependent DnaK propensity score for wild-type CI2. (**b**) Variation in DnaK binding energy for single point mutations in the major DnaK binding region of CI2. (**c**) Distributions of changes in DnaK binding scores of CI2 variants relative to the score of wild-type CI2, for all 71 initially selected variants (black), those variants that failed to be purified (red), and those that were purified and used for stability measurements (green).
