# Supplementary material for: Synergistic stabilization of a double mutant in chymotrypsin inhibitor 2 from a library screen in E. coli
Source: Commun Biol. 2021 Aug 18;4:980. doi: 10.1038/s42003-021-02490-7 (PMC8373930; doi:10.1038/s42003-021-02490-7)
Supplement: Supplementary file 3 — Description of Additional Supplementary Files [file 42003_2021_2490_MOESM3_ESM.pdf]

Description of Additional Supplementary Files

File Name: Supplementary Data 1
Description: zip.file with FACS data used for Fig 1a

File Name: Supplementary Data 2
Description: excel-file with source data for Fig 1b

File Name: Supplementary Data 3
Description: excel-file with source data for Fig 1c

File Name: Supplementary Data 4
Description: excel-file with source data for Fig 2

File Name: Supplementary Data 5
Description: excel-file with source data for Fig 3a

File Name: Supplementary Data 6
Description: excel-file with source data for Fig 3b
