# Supplementary material for: Synergistic stabilization of a double mutant in chymotrypsin inhibitor 2 from a library screen in E. coli
Source: Commun Biol. 2021 Aug 18;4:980. doi: 10.1038/s42003-021-02490-7 (PMC8373930; doi:10.1038/s42003-021-02490-7)
Supplement: Supplementary file 10 — Reporting Summary [file 42003_2021_2490_MOESM10_ESM.pdf]

nature portfolio

Corresponding author(s): Kaare Teilum

Last updated by author(s): Jul 15, 2021

Reporting Summary

Nature Portfolio wishes to improve the reproducibility of the work that we publish. This form provides structure for consistency and transparency in reporting. For further information on Nature Portfolio policies, see our Editorial Policies and the Editorial Policy Checklist.

Statistics

For all statistical analyses, confirm that the following items are present in the figure legend, table legend, main text, or Methods section.

n/a	Confirmed	
☒	☐	The exact sample size (*n*) for each experimental group/condition, given as a discrete number and unit of measurement
☒	☐	A statement on whether measurements were taken from distinct samples or whether the same sample was measured repeatedly
☒	☐	The statistical test(s) used AND whether they are one- or two-sided<br>*Only common tests should be described solely by name; describe more complex techniques in the Methods section.*
☒	☐	A description of all covariates tested
☒	☐	A description of any assumptions or corrections, such as tests of normality and adjustment for multiple comparisons
☒	☐	A full description of the statistical parameters including central tendency (e.g. means) or other basic estimates (e.g. regression coefficient) AND variation (e.g. standard deviation) or associated estimates of uncertainty (e.g. confidence intervals)
☒	☐	For null hypothesis testing, the test statistic (e.g. *F*, *t*, *r*) with confidence intervals, effect sizes, degrees of freedom and *P* value noted<br>*Give P values as exact values whenever suitable.*
☒	☐	For Bayesian analysis, information on the choice of priors and Markov chain Monte Carlo settings
☒	☐	For hierarchical and complex designs, identification of the appropriate level for tests and full reporting of outcomes
☒	☐	Estimates of effect sizes (e.g. Cohen's *d*, Pearson's *r*), indicating how they were calculated

Our web collection on statistics for biologists contains articles on many of the points above.

Software and code

Policy information about availability of computer code

Data collection	Fluorescence temperature scans: Prometheus PR.ThermalControl, ver. 2.1.1 (NanoTemper)<br>X-ray crystallographic data collection: autoProc, ver 1.0.5 (Global Phasing)
Data analysis	Analysis of protein unfolding: ProteinUnfolding2D, (https://github.com/KULL-Centre/ProteinUnfolding2D)<br>Calculation of ddG: FoldX, ver. 4 (http://foldxsuite.crg.eu)<br>Calculation of ddG: Rosetta, ver 3.6 (https://www.rosettacommons.org)<br>Calculation of ddG from sequence: https://github.com/KULL-Centre/papers/tree/master/2021/CI2-Hamborg-et-al<br>Prediction of DnaK binding: Limbo (https://limbo.switchlab.org)<br>Analysis of X-ray crystallographic data: CCP4i, ver. 6.5.019 (https://www.ccp4.ac.uk)<br>Analysis of X-ray crystallographic data: coot, ver. 0.9.2

For manuscripts utilizing custom algorithms or software that are central to the research but not yet described in published literature, software must be made available to editors and reviewers. We strongly encourage code deposition in a community repository (e.g. GitHub). See the Nature Portfolio guidelines for submitting code & software for further information.

Data

Policy information about availability of data

All manuscripts must include a data availability statement. This statement should provide the following information, where applicable:

- Accession codes, unique identifiers, or web links for publicly available datasets
- A description of any restrictions on data availability
- For clinical datasets or third party data, please ensure that the statement adheres to our policy

The atomic coordinates and structure factors for the structures presented in this work are deposited at the Protein Data Bank (https://www.wwPDB.org) under accession numbers 7A1H, 7A3M, 7AOK and 7AON. Source data for graphs are available as Supplementary Data. Other data included in the figures and that support the findings of this study are available from the corresponding author upon reasonable request.

Field-specific reporting

Please select the one below that is the best fit for your research. If you are not sure, read the appropriate sections before making your selection.

[x] Life sciences [ ] Behavioural & social sciences [ ] Ecological, evolutionary & environmental sciences

For a reference copy of the document with all sections, see nature.com/documents/nr-reporting-summary-flat.pdf

Life sciences study design

All studies must disclose on these points even when the disclosure is negative.

Sample size	The stability analysis was performed on 6-16 samples. We previously performed a systematic analysis of the minimal number of samples required for a reliable analysis and found that five samples were sufficient for getting reliable results.
Data exclusions	Sequences including internal stop codons were excluded from further analysis. We excluded variants that could not be expressed and purified for further analysis. We also excluded variant that did not give useful equilibrium unfolding data from the analysis.
Replication	The stability were replicated 2-5 times. As common, the crystal structures were only repeated once.
Randomization	This is a biophysical study with no biological variation.
Blinding	This is a biophysical study and blinding is not relevant.

Reporting for specific materials, systems and methods

We require information from authors about some types of materials, experimental systems and methods used in many studies. Here, indicate whether each material, system or method listed is relevant to your study. If you are not sure if a list item applies to your research, read the appropriate section before selecting a response.

Materials & experimental systems

n/a	Involved in the study	
[x]	[ ]	Antibodies
[x]	[ ]	Eukaryotic cell lines
[x]	[ ]	Palaeontology and archaeology
[x]	[ ]	Animals and other organisms
[x]	[ ]	Human research participants
[x]	[ ]	Clinical data
[x]	[ ]	Dual use research of concern

Methods

n/a	Involved in the study	
[x]	[ ]	ChIP-seq
[ ]	[x]	Flow cytometry
[x]	[ ]	MRI-based neuroimaging

March 2021

Flow Cytometry

Plots

Confirm that:

- [x] The axis labels state the marker and fluorochrome used (e.g. CD4-FITC).
- [x] The axis scales are clearly visible. Include numbers along axes only for bottom left plot of group (a 'group' is an analysis of identical markers).
- [x] All plots are contour plots with outliers or pseudocolor plots.
- [x] A numerical value for number of cells or percentage (with statistics) is provided.

Methodology

Sample preparation	E. coli transformed with a folding sensor library of CI2
Instrument	BD FACS-Aria SORP cell sorter
Software	BD FACSDiva
Cell population abundance	Sorting was not used to isolate cell types, but to select a fraction of a library with a continuous change in GFP signal
Gating strategy	Cells expressing variants of wild type CI2 were sorted in two successive rounds for high GFP fluorescence, defined as the upper approximately 1 % of the GFP signal. In the same way, the library made in the I57A background was sorted for the lower approximately 1 % of the GFP signal.

- [x] Tick this box to confirm that a figure exemplifying the gating strategy is provided in the Supplementary Information.

March 2021
